# Electronic control in random access analysers

**DOI:** 10.1155/S1463924688000379

**Published:** 1988

**Authors:** A. Truchaud, C. A. Burtis

**Affiliations:** 1 Biochimie Hôspital de Meaux Meaux F77104 France; 2 Chemical Technology and Health Divisions Oak Ridge National Laboratory Oak Ridge Tennessee 37831 USA


Journal of Automatic Chemistry Vol. 10, No. 4 (October–December 1988), pp. 179–180

### Electronic control in random access analysers

**A. Truchaud**
*Biochimie, Hôpital de Meaux, F77104 Meaux, France*

**and C. A. Burtis**
*Chemical Technology and Health Divisions, Oak Ridge National Laboratory, Tennessee, Oak Ridge, 37831, USA*

*Improvements in microchip technology have produced a new breed of computerized laboratory analysers. Modern analysers have both self-acting and self-regulating mechanisms. Automatic self-checking diagnostics allow preventive service and repair. An instrument can be implemented through changes in software. Chemometrics help to check reagent integrity, validate calibration, and quality control. Computer-assisted interpretation is just beginning to be a possibility.*

*The computer revolution has resulted in a new breed of instruments for laboratory analyses. This paper discusses the impact of electronics on process control, diagnostics, instrument upgrades and chemometrics.*

### Process control

It would take hours for a human being to choose the best way to operate different panels of parameters; random access analysers do so immediately. Analysers like the Technicon Autoanalysers, were first *mechanized*, now they are *automated* with self-acting and self-regulating mechanisms.

In old instruments, a central processor was used to carry out all functions with real constraints placed on the software. Decentralized control provides much more control and security, and connection with other computers is easier – see figure 1.

### Diagnostics

Automatic self-checking diagnostics on start up or during operation provide assurance on electrochemical, memory and computational functions. Diagnosis can be requested by the operator for preventive service or repair. Instrument repair, often by replacement of a circuit board is usually made easier with system diagnostics and downtime is minimized.

### *Upgrading instruments*

All the functions are software controlled, i.e. by read only memory (ROM) chips, programmed by the manufacturer. As a consequence, changes in the operation and capabilities of an instrument can now be implemented through changes in software (via a disk), a memory board or a microchip on a board. Examples are data for new parameters, new algorithms for calibration curves, accelerating a process to increase throughput, and changing the operation of a robotic part of the system.

### *Chemometrics*

Chemometrics is the application of mathematical and statistical methods to chemistry. Modern systems are able to acquire, store, process and manage large data sets in real time by using a variety of sophisticated algorithms. Chemometrics uses such techniques as advanced statistics, imaging, optimization and control, artificial intelligence, and computer-assisted interpretation.

It is appropriate to emphasize the control of the measurements:

For a single chemical reaction, curves can be fitted to pre-established algorithms, providing checks on reagent integrity. This gives better linearity and fewer re-runs.

Limits for slope and intercept of a linear calibration can be memorized – see figure 2.

Quality control programs can be included in the software with acceptable limits.

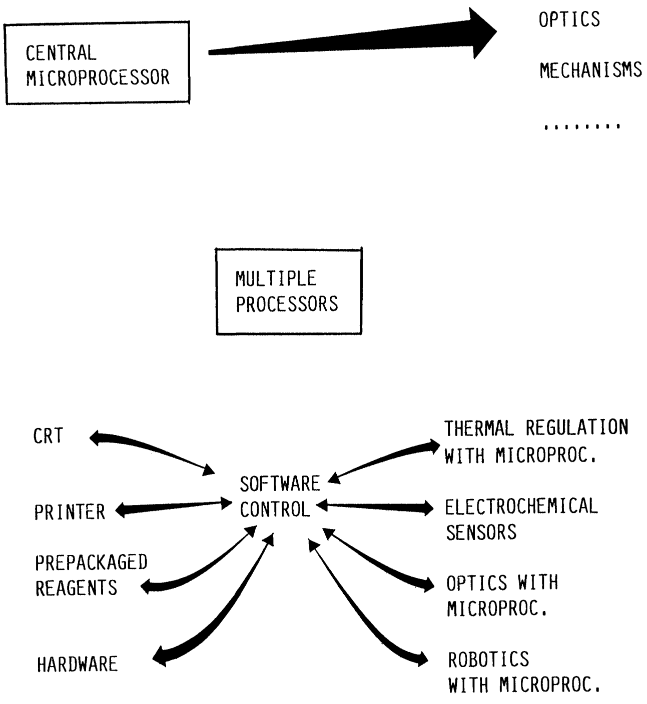

*Figure 1. Evolution of microprocessor control in random access analysers.*

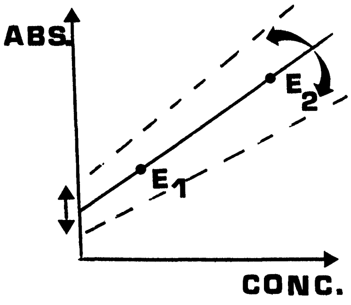

*Figure 2. Validation of the calibration step.*

### Computer-assisted interpretation

Computer-assisted interpretation in clinical chemistry presently includes interpretation of a patient result compared to the range for a given population (age, sex, illness); interpretation of a profile of parameters for one patient; and the suggestion of a complementary analysis.

### Conclusion

Microchip technology will have an increasing impact on the laboratory with the advent of fully automated analytical systems. Therefore there must be a significant effect on the way laboratories are organized and operated.

### EUROANALYSIS VII

*26–31 August, 1990: Vienna, Austria*

This all-European Conference will emphasize methodological developments and especially the role of analytical chemistry for problem solving in major areas of science. Additionally, special sessions discussing highly relevant topics, like COBAC (Computer Based Analytical Chemistry), will be scheduled. Furthermore a large exhibition of instruments, reagents and literature, as well as an attractive social programme will be organized.

*For registration and further information contact the Secretary General, Professor Dr M. Grasserbauer, c/o INTERCONVENTION, Austria Center Vienna, A-1450 Vienna, Austria.*

### NOTES FOR AUTHORS

*Journal of Automatic Chemistry* covers all aspects of automation and mechanization in analytical, clinical and industrial environments. The *Journal* publishes original research papers; short communications on innovations, techniques and instrumentation, or current research in progress; reports on recent commercial developments; and meeting reports, book reviews and information on forthcoming events. All research papers are refereed.

### Manuscripts

Two copies of articles should be submitted. All articles should be typed in double spacing with ample margins, on one side of the paper only. The following items should be sent: (1) a title-page including a brief and informative title, avoiding the word 'new' and its synonyms; a full list of authors with their affiliations and full addresses; (2) an abstract of about 250 words; (3) the main text; (4) appendices (if any); (5) references; (6) tables, each table on a separate sheet and accompanied by a caption; (7) illustrations (diagrams, drawings and photographs) numbered in a single sequence from 1 upwards and with the author's name on the back of every illustration; captions to illustrations should be typed on a separate sheet. Papers are accepted for publication on condition that they have been submitted only to this *Journal*.

### References

References should be indicated in the text by numbers following the author's name, i.e. Skeggs [6]. In the reference section they should be arranged thus:

to a journal

Manks, D. P., *Journal of Automatic Chemistry*, **3** (1981), 119.

to a book

Malmstadt, H. V., in *Topics in Automatic Chemistry*, Ed. Stockwell, P. B. and Foreman, J. K. (Horwood, Chichester, 1978), p. 68.

### Illustrations

Original copies of diagrams and drawings should be supplied, and should be drawn to be suitable for reduction to the page or column width of the *Journal*, i.e. to 85 mm or 179 mm, with special attention to lettering size. Photographs may be sent as glossy prints or as negatives.

### Proofs and offprints

The principal or corresponding author will be sent proofs for checking and will receive 50 offprints free of charge. Additional offprints may be ordered on a form which accompanies the proofs.

*Manuscripts should be sent to Dr P. B. Stockwell, P.S. Analytical Ltd, Arthur House, Cray Avenue, Orpington, Kent BR5 3TR, UK.*

